# A Critical Intervention for Sustainable Health: Climate Change Awareness Among Nurse Managers

**DOI:** 10.1155/jonm/2150047

**Published:** 2026-04-30

**Authors:** Emine Ekici, Seda Baykara Mat, Ozan Ozkol, Fikriye Isık

**Affiliations:** ^1^ School of Nursing, Maltepe University, Istanbul, Türkiye, maltepe.edu.tr; ^2^ Faculty of Health Sciences, Department of Nursing, Beykent University, Istanbul, Türkiye, beykent.edu.tr; ^3^ Kartal Dr. Lütfi Kırdar Training and Research Hospital, Istanbul, Türkiye

**Keywords:** awareness, climate change, nurse managers, nursing education, sustainability

## Abstract

**Purpose:**

This study aimed to evaluate the effectiveness of a nurse‐led educational program designed to improve nurse managers’ awareness, knowledge, and attitudes regarding climate change and its health impacts.

**Background/Introduction:**

Climate change is one of the most urgent global public health challenges, jeopardizing key determinants of health such as air quality, access to safe drinking water, food security, and adequate housing. As frontline healthcare providers, nurses are uniquely positioned to identify environmental health risks and promote climate‐resilient healthcare practices. Despite this critical role, evidence suggests that nurses’ awareness and preparedness for climate‐related health threats are insufficient. Therefore, strengthening climate literacy among nurse leaders is essential to enhance adaptation capacity in health systems.

**Methods:**

This study used a pretest–posttest experimental design with hospital‐level randomization and included 108 nurse managers working in two public hospitals in Istanbul. Participants were randomly assigned to intervention and control groups. The study was conducted between March and June 2025, with data collection carried out between April and May 2025. Data were collected using a Descriptive Information Form and the Climate Change Awareness Scale (CCAS). The intervention group received 90 min of face‐to‐face training, including theoretical content, case‐based learning, and interactive assessment, whereas the control group received a 90‐min lecture after data collection on climate change and its health impacts, followed by a brief question‐and‐answer session. Measurements were taken at baseline, immediately after the intervention, and 1 month later. Data were analyzed using Friedman and Wilcoxon signed‐rank tests.

**Results/Findings:**

The intervention group demonstrated significant improvements in total and subscale CCAS scores at the postintervention time point compared with baseline and the control group (*p* < 0.05). Although a slight decrease was observed at the 1‐month follow‐up, scores remained higher than pretest levels. In the control group, although small differences were observed in certain subscales in the between‐group comparisons, no statistically significant within‐group changes were observed between the pretest, posttest, and follow‐up scores.

**Discussion:**

The findings suggest that structured and nurse‐led climate training for nurse managers has the potential to strengthen climate‐related awareness and preparedness capacity.

**Conclusion:**

Even short‐term training increases nurse managers’ awareness of climate change and health.

**Trial Registration:** ClinicalTrials.gov identifier: NCT06905548

## 1. Introduction

Climate change is a global public health issue that adversely affects the fundamental determinants of health, including clean air, safe drinking water, food security, and adequate shelter [1]. The Lancet Countdown on Health and Climate Change (2021) reported an increase in the adverse health impacts of climate change, emphasizing the urgent need for preventive action [2]. Moreover, the World Health Organization projects approximately 250,000 additional deaths between 2030 and 2050 due to climate‐related health problems such as undernutrition, malaria, and diarrheal diseases [3]. Given these risks, climate change represents a critical area of concern that demands the attention of all health professionals [4]. Health systems must not only continue their essential functions of protecting and promoting population health but also develop strategies to mitigate their own environmental impacts. In this context, nurseswho are frontline providers of patient careneed to understand the health consequences of climate change and cultivate awareness of its implications [5, 6]. In addition to studies emphasizing the significance of nursing in addressing health challenges posed by climate change, international organizations such as the International Council of Nurses (ICN) have highlighted nurses’ pivotal roles in this process [4, 7–9]. However, research also indicates that nurses often lack sufficient knowledge and fail to respond adequately to the issue of climate change [10–12].

The first step in assessing the capacity to cope with climate‐related challenges is to determine the level of awareness. Identifying this awareness provides the foundation for developing positive attitudes and behaviors toward climate action. The literature demonstrates that educational programs implemented across various populations have been effective in enhancing awareness of climate change [13, 14].

Nurses represent a professional group with a fundamental role in mitigating the health consequences of climate change [7]. Therefore, nurses’ awareness of climate change is considered a key element in ensuring the effective delivery of health services, protecting public health, and supporting the sustainability of health systems [15].

In a study examining the relationships among nurses’ knowledge and behaviors related to climate change, specifically awareness, concern, motivation, and behaviors at home and in the workplace, motivation, concern, and workplace behaviors were identified as factors influencing attitudes toward environmental sustainability [16]. Similarly, a web‐based educational intervention designed to enhance primary care nurses’ climate change awareness, activism, and environmentally friendly behaviors demonstrated significant improvements in participants’ knowledge and behaviors following the training [17]. A study examining the factors influencing nurses’ behaviors to mitigate the effects of climate change emphasized that access to environmental information enhances self‐efficacy. Nurses who integrate up‐to‐date environmental knowledge into their practice can take a leading role in environmental initiatives [18]. Increasing nurses’ capacity to reduce the impacts of climate change requires education in environmental sustainability and leadership, both of which are considered critically important [7, 19]. Nurse managers, in particular, have the potential to influence nurses’ proenvironmental attitudes and behaviors positively. Educators and administrators should identify the barriers to these behaviors and develop supportive workplace cultures and sustainable policies to foster change [16]. Enhancing nurse managers’ awareness of climate change is essential not only for improving the effectiveness of healthcare delivery but also for minimizing the negative environmental and social consequences of healthcare services. Therefore, this study implemented an educational program aimed at improving nurse managers’ awareness of climate change.

## 2. Methods

### 2.1. Research Hypothesis

H_1_: The climate change awareness levels of nurse managers in the intervention group who received climate change awareness training will be significantly higher after the training compared with before the training and compared with the control group.

### 2.2. Research Design

This study used a pretest–posttest experimental design with hospital‐level randomization to evaluate the effect of climate change awareness training on the awareness levels of nurse managers. This study, titled “Climate Change Awareness,” was conducted between 25 March, 2025, and subsequent follow‐up assessments. The research design was selected to evaluate the effect of the educational intervention by comparing pre‐ and postintervention measurements between the intervention and control groups.

### 2.3. Participants and Sample

The study population consisted of nurse managers working at two similar city hospitals in Istanbul, Türkiye (71 and 58 nurse managers, respectively; total *N* = 129). These hospitals were selected because of their similar service capacities, bed numbers, patient volumes, and organizational structures. Both hospitals operate under the Istanbul Provincial Health Directorate and are public city hospitals with very similar organizational frameworks, nursing service management hierarchies, and job descriptions.

This similarity ensured that the intervention and control groups were homogeneous in terms of organizational and structural characteristics. Furthermore, the similar number of nurse managers working in both hospitals allowed for the creation of two equivalent groups suitable for comparison. A census method, which aims to include the entire population, was used as the sampling approach. Since the number of nurse managers in both hospitals was limited, it was deemed appropriate to reach the entire population [20]. However, since participation was voluntary, the final sample size was determined based on actual participation rates. Therefore, a power analysis was performed to assess the adequacy of the obtained sample size. A priori power analysis using GPower 3.1.9.7 showed that at least 45 participants in each group were needed to detect a large effect (*d* = 0.79) with 95% power at *α* = 0.05 [21, 22]. Although the final sample consisted of 35 participants in each group, a post hoc sensitivity analysis showed that the study retained approximately 80% power to detect effects of *d* ≥ 0.67, which is an acceptable value for behavioral and educational research.

### 2.4. Inclusion Criteria

• Actively employed in a managerial position at one of two large public hospitals in Istanbul, Türkiye;
• Expected to remain in their current position at the same institution throughout the study period;
• Had not previously participated in an extensive training program (e.g., longer than 8 h) on climate change, environmental management, or sustainability;
• Voluntarily agreed to participate in the study and signed the informed consent form;
• Were able to fully participate in the training process and in both pretest and posttest assessments.

### 2.5. Determination of Intervention and Control Groups

The nurse managers who participated in the study were employed at two public hospitals in Istanbul with comparable institutional characteristics. To ensure comparability between groups, the hospitals where the participants worked were randomly assigned as the intervention and control groups.

The randomization process was carried out by an independent biostatistics expert who was not part of the research team. The “Random Number Generator” tool available at https://www.random.org/ was used to perform the random assignment. As a result, one hospital was designated as the intervention group and the other as the control group.

**FIGURE 1:**
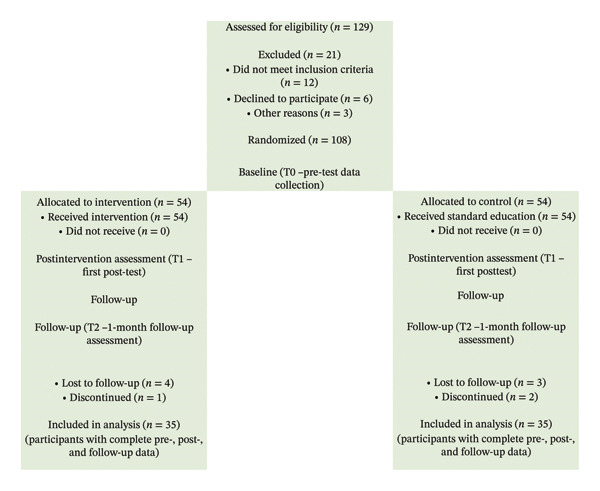
CONSORT flow diagram of the study (2025). CONSORT flow diagram illustrating participant allocation, intervention timing, and data collection points (T0: pretest (April 15, 2025), T1: postintervention (April 15, 2025), T2: 1‐month follow‐up (May 15, 2025)).

Nurse managers in the intervention group received the planned nurse‐led climate change awareness training program developed for this study. The control group did not receive the intervention during the study period; training was provided only after all data collection, and no data were collected thereafter. This approach was chosen to minimize selection bias, enhance group comparability, and ensure the reliable evaluation of the training program’s effect. Each stage of this randomized controlled trial was conducted in accordance with the Consolidated Standards of Reporting Trials (CONSORT) guidelines, as illustrated in Figure 1.

### 2.6. Minimization of Bias

Several methodological strategies were implemented to reduce the potential impact of bias in this study. First, randomization was performed at the hospital level, with the two hospitals randomly assigned to the intervention and control conditions by an independent biostatistics expert using https://www.random.org/. This approach was used to minimize selection bias at the institutional level; however, individual participant randomization was not performed. Predefined inclusion and exclusion criteria were used during participant recruitment to ensure sample homogeneity. In addition, data were collected using standardized instruments, namely, the Descriptive Information Form and the Climate Change Awareness Scale (CCAS). As both instruments have established validity and reliability, the risk of measurement bias was minimized.

The educational intervention was delivered by the same researcher to all participants, using identical content, duration, and conditions. This ensured consistency in implementation and prevented performance bias. During data coding and analysis, participant groups were identified by numeric codes rather than names, following a blinding procedure to reduce detection bias. Finally, data confidentiality was strictly maintained. Participation in the study was entirely voluntary, and ethical principles were rigorously followed to ensure that participants experienced no pressure or coercion when providing their responses.

### 2.7. Data Collection Instruments

Data were collected using two instruments: the Descriptive Information Form and the CCAS.

#### 2.7.1. Descriptive Information Form

This form was developed by the researchers to identify participants’ sociodemographic characteristics. It consists of 12 items covering variables such as gender, age, educational background, managerial position, years of professional experience, and previous participation in any training related to climate change.

#### 2.7.2. CCAS

CCAS was developed by Ataklı and Kuran [23]. It is a five‐point Likert‐type instrument consisting of 52 items and 5 subscales: (1) awareness of climate change, (2) perception of the problem, (3) knowledge of causes, (4) level of concern, and (5) behaviors and expectations regarding policies.

Possible scores range from 52 to 260, with higher scores indicating greater awareness of climate change. The Cronbach’s alpha coefficient of the scale was reported as 0.92, demonstrating excellent internal consistency. Permission to use the scale was obtained from the corresponding author via e‐mail. The overall Cronbach’s alpha coefficient of the scale was found to be 0.987. When the subdimensions were analyzed, the Cronbach’s alpha values were calculated as 0.888 for the climate change awareness subdimension, 0.944 for the perception of the problem subdimension, 0.974 for the knowledge of causes of climate change subdimension, 0.981 for the climate change concern subdimension, and 0.987 for the expectations from behaviors and policies subdimension. These findings indicate that both the overall scale and all subdimensions have a high level of internal consistency.

### 2.8. Data Collection Procedure

The data collection process was structured and conducted sequentially according to the following stages:

1. A structured demographic information form was administered to determine the participants’ demographic and identifying characteristics.
2. Hospitals were randomly assigned to the intervention and control groups using a computerized randomization method.
3. A pilot study was conducted to evaluate the comprehensibility and applicability of the case scenario using data collection tools.
4. Preintervention CCAS pretest data were collected from both groups.
5. A case‐based training program was implemented for the intervention group.
6. An evaluation session was held with the intervention group after the case study.
7. Postintervention data from CCAS (first measurement/posttest) were collected from both groups.
8. To assess the long‐term retention of learning outcomes, CCAS data were collected from both groups 1 month after the intervention as a follow‐up measurement (second measurement).
9. After all measurements related to the study were completed, in line with ethical responsibilities, the control group was provided with training covering the same theoretical content as the intervention group; no data were collected at this stage.

### 2.9. Intervention

During the intervention phase of the study, a comprehensive educational program was implemented to enhance nurse managers’ awareness of climate change. The program was designed to integrate both theoretical knowledge and active learning strategies, encouraging participants’ engagement throughout the process.

The training was conducted face‐to‐face by the researchers and lasted approximately 90 min. The training content and duration were structured using a case‐based and interactive approach, aligned with the intended learning outcomes. The 90‐min duration was deliberately chosen to accommodate the administrative responsibilities and time constraints of nurse managers, while providing a focused and actionable intervention that could be integrated into routine clinical practice without disrupting workflow. In the first part of the session, participants received information on the health impacts of climate change, the environmental responsibilities of the health sector, and the leadership roles of nurses in addressing these issues. Theoretical content was supported with visual materials, including slide presentations, infographics, and short videos. The content, instructional methods, and duration of the climate change awareness training are presented in Table 1.

**TABLE 1:** Structure, content, and duration of the climate change and health awareness training program.

Training component	Content	Method	Duration (min)
Theoretical instruction	Core concepts and drivers of climate change; relationship between climate change and health; health impacts of extreme weather events, air pollution, and water and food insecurity; role of nurse managers in addressing climate change within healthcare systems	Interactive lecture, question‐answer	30
Case‐based learning and group discussions	Hospital preparedness for extreme heat events; community health risks following floods; sustainable healthcare practices and climate adaptation strategies	Case analysis, structured group discussion	40
Interactive evaluation and feedback	Reinforcement of key concepts; collection of participant feedback; overall evaluation of the training	Online interactive tools (e.g., Mentimeter), discussion	20
Total			90

In the second part, case‐based learning activities were conducted to promote active participation. These case scenarios illustrated potential climate‐related challenges in healthcare settings such as energy use, waste management, and preparedness, and participants were asked to propose practical solutions. This approach aimed to strengthen both individual and managerial levels of awareness and problem‐solving skills.

At the end of the training session, an online platform was used to reinforce learning and increase participant motivation. This interactive online assessment tool allowed participants’ knowledge levels to be evaluated in an engaging and enjoyable learning environment.

The same measurement instruments were administered before and after the training to assess changes in awareness levels. The training content and implementation steps were standardized across both hospitals to ensure methodological consistency.

### 2.10. Data Collection

After obtaining the necessary institutional approvals from the Provincial Health Directorate, the researchers met with the nurse educators of the participating hospitals to provide detailed information about the purpose and content of the study. The nurse educators then informed the nurse managers through existing digital communication channels and created lists of those who voluntarily agreed to participate. Data collection forms and the preliminary application of the case study were conducted on 30 March, 2025, with 8 participants to assess clarity and feasibility. Participants in the pilot study were not included in the main study sample. The intervention and control groups were determined based on hospital assignment. Separate digital communication groups were established for each hospital, through which information regarding the training dates, locations, and participation procedures was shared.

Prior to the intervention, participants completed their baseline (pretest) assessments. To assess the retention of learning outcomes, the same assessment tool was administered again immediately after the completion of the training program and 4 weeks later. All data were collected by researchers through face‐to‐face interviews.

### 2.11. Training Provided to the Control Group

After the completion of all data collection procedures, a training session was provided to the control group in accordance with ethical considerations. This training was designed to ensure that participants in the control group were not disadvantaged by their nonexposure to the intervention during the study period. The session was delivered face‐to‐face by the researchers and consisted solely of a didactic, lecture‐based format. The content included fundamental information on climate change, its health impacts, and the role of healthcare professionals, similar in scope to the theoretical component of the intervention group. No interactive methods such as case‐based learning, group discussion, or assessment activities were used in this session. The training lasted approximately 90 min and was conducted using slide presentations as the primary instructional material. No data were collected from the control group following this training.

### 2.12. Ethical Approval

This study was conducted in accordance with the principles of the Declaration of Helsinki, revised in Brazil in 2013. Ethical approval was obtained from Maltepe University Non‐Interventional Scientific Research Ethics Committee (Decision number: [2025/03‐08], Date: 2025/03/08). In addition, written permission was received from the institution where the research was conducted. Prior to data collection, all participants were provided with detailed information about the purpose, content, potential risks, and benefits of the study. Participation was entirely voluntary, and participants were informed of their right to withdraw from the study at any time without penalty. Written informed consent was obtained from all individuals who agreed to participate. After all measurements related to the study were completed, in accordance with ethical responsibilities, the control group was provided with training consisting of two sessions covering the same theoretical content as the intervention group; no data were collected at this stage.

### 2.13. Statistical Analysis

All statistical analyses were performed using IBM SPSS Statistics for Windows, Version 25.0. Descriptive statistics, including frequency, percentage, mean, and standard deviation, were used to summarize the participants’ demographic and study‐related characteristics. The Shapiro–Wilk test was applied to assess the normality of data distribution. For variables that met the assumption of normality, independent samples *t*‐tests were conducted to compare differences between the intervention and control groups, whereas paired samples *t*‐tests were used to evaluate within‐group (pretest and posttest) changes. For nonnormally distributed variables, nonparametric tests such as the Mann–Whitney *U* test and Wilcoxon signed‐rank test were employed. Additionally, Pearson’s correlation analysis was performed to examine relationships between continuous variables. All analyses were conducted with a 95% confidence interval, and statistical significance was set at *p* < 0.05. All survey forms were designed to ensure complete anonymity and confidentiality of participant information. The collected data were stored securely and used exclusively for research purposes. The researchers are committed to maintaining participant privacy, protecting personal rights, and ensuring that all data are kept under their supervision. Permission to use the scales employed in the study was also obtained from the respective authors.

## 3. Results

The Shapiro–Wilk test was conducted to assess the assumption of normality. The results indicated that the overall and subscale scores of the measurement instrument were not normally distributed. Therefore, nonparametric statistical techniques were employed in the data analysis.

The Cronbach’s alpha coefficients for CCAS and its subscales ranged from 0.888 to 0.987, indicating a high level of internal consistency and reliability. In the intervention group, most nurse managers were female (47.1%) and held the position of charge nurse (34.3%); 24.3% had completed graduate education. Additionally, 22.9% had 16–20 years of professional experience, and 27.1% worked in internal medicine units. In the control group, most participants were female (45.7%) and held a graduate degree (30%), while 28.6% served as charge nurses. Among all participants, 47.1% had not received any prior training on climate change, 40% had participated in an awareness program, and 14.3% reported gaining information through media sources. The mean age of participants across both groups was 36.25 ± 7.55 years. On a self‐reported scale ranging from 0 to 10, the average level of knowledge about climate change was found to be 4.42 ± 2.66.

No statistically significant differences were found between the intervention and control groups in terms of gender, educational level, job position, years of professional experience, clinical unit, prior participation in climate‐related or awareness training, or self‐reported knowledge of climate change. These findings indicate that the groups were homogeneous across these variables. However, a statistically significant difference was found between the groups in terms of age; this indicates that the groups are not homogeneous with respect to this variable.

In the intervention group, the mean total score of CCAS increased from 203.40 ± 53.95 at the pretest to 243.06 ± 20.87 at the posttest, and was measured as 231.91 ± 32.84 at the follow‐up assessment. In the control group, the corresponding scores were 200.89 ± 43.60, 220.43 ± 29.15, and 219.43 ± 32.71, respectively (Table 2).

**TABLE 2:** Distribution of the CCAS and its subdimensions according to pretest, posttest, and remeasurement scores.

Groups	Scales and subdimensions	Mean	SD	Med.	Min.	Max.
Intervention group	According to pretest scores					
	CCAS total	203.40	53.95	220.00	60.00	260.00
	Awareness of climate change	31.23	7.73	32.00	12.00	45.00
	Perception of the problem	19.40	5.61	20.00	5.00	25.00
	Knowledge of the causes of climate change	35.00	10.95	36.00	9.00	45.00
	Concern about climate change	44.83	13.22	50.00	11.00	55.00
	Behaviors and expectations regarding policies	72.94	21.41	80.00	18.00	90.00
	According to posttest scores					
	CCAS total	243.06	20.87	250.00	193.00	260.00
	Awareness of climate change	40.40	4.49	42.00	32.00	45.00
	Perception of the problem	23.63	2.20	25.00	19.00	25.00
	Knowledge of the causes of climate change	42.11	4.19	45.00	33.00	45.00
	Concern about climate change	52.17	4.74	55.00	40.00	55.00
	Behaviors and expectations regarding policies	84.74	7.64	90.00	67.00	90.00
	According to follow‐up scores					
	CCAS total	231.91	32.84	242.00	88.00	260.00
	Awareness of climate change	36.94	5.94	37.00	12.00	45.00
	Perception of the problem	22.29	4.03	25.00	10.00	25.00
	Knowledge of the causes of climate change	40.60	6.44	45.00	18.00	45.00
	Concern about climate change	49.94	7.48	53.00	17.00	55.00
	Behaviors and expectations regarding policies	82.14	11.37	87.00	31.00	90.00

Control group	According to pretest scores					
	CCAS total	220.43	29.15	236.00	148.00	252.00
	Awareness of climate change	34.17	5.60	34.00	14.00	43.00
	Perception of the problem	19.89	4.84	20.00	8.00	25.00
	Knowledge of the causes of climate change	38.40	6.39	39.00	23.00	45.00
	Concern about climate change	48.31	8.37	53.00	19.00	55.00
	Behaviors and expectations regarding policies	79.66	10.44	83.00	54.00	90.00
	According to posttest scores					
	CCAS total	200.89	43.60	208.00	153.00	260.00
	Awareness of climate change	35.34	6.46	36.00	27.00	45.00
	Perception of the problem	17.09	7.14	20.00	8.00	25.00
	Knowledge of the causes of climate change	33.80	8.53	36.00	24.00	45.00
	Concern about climate change	38.34	15.29	44.00	19.00	55.00
	Behaviors and expectations regarding policies	76.31	9.29	73.00	54.00	90.00
	According to follow‐up scores					
	CCAS total	219.43	32.71	231.00	152.00	260.00
	Awareness of climate change	35.83	6.21	35.00	22.00	45.00
	Perception of the problem	19.69	4.24	20.00	9.00	25.00
	Knowledge of the causes of climate change	37.51	6.22	37.00	25.00	45.00
	Concern about climate change	48.26	7.19	50.00	32.00	55.00
	Behaviors and expectations regarding policies	78.14	12.67	82.00	45.00	90.00

Abbreviations: CCAS, Climate Change Awareness Scale; Mean, arithmetic mean; Med, median; SD, standard deviation.

When the pretest mean scores of CCAS and its subscales were compared between the intervention and control groups, no statistically significant differences were found, indicating that the two groups were comparable at baseline (Table 3). At the posttest, the mean total and subscale scores of the intervention group were significantly higher than those of the control group (Table 3).

**TABLE 3:** Comparison of the CCAS general and subdimension scores of the intervention and control groups.

Scales and subdimensions	Intervention group (*n*: 35)			Control group (*n*: 35)			Statistical analysis
	Mean	Sd	Med.	Mean	Sd	Med.	
a. Total CCAS: pretest	203.40	53.95	220.00	220.43	29.15	236.00	*U*: 522.5 *p*: 0.290
b. Total CCAS: posttest	243.06	20.87	250.00	200.89	43.60	208.00	*U*: 302.5 *p*: 0.000
c. Total CCAS: follow‐up	231.91	32.84	242.00	219.43	32.71	231.00	*U*: 446.5 *p*: 0.050
Statistical analysis/intragroup comparison	a‐b = *Z*: −3.89, *p*: 0.000 a–c = *Z*: −2.94, *p*: 0.003			a‐b = *Z*: −2.15, *p*: 0.032 a–c = *Z*: −0.068, *p*: 0.945			

a. Awareness of climate change: pretest	31.23	7.73	32.00	34.17	5.60	34.00	*U*: 449.0 *p*: 0.054
b. Awareness of climate change: posttest	40.40	4.49	42.00	35.34	6.46	36.00	*U*: 344.0 *p*: 0.001
c. Awareness of climate change: follow‐up	36.94	5.94	37.00	35.83	6.21	35.00	*U*: 503.5 *p*: 0.199
Statistical analysis/intragroup comparison	a‐b = *Z*: −4.64, *p*: 0.000 a–c = *Z*: −3.53, *p*: 0.000			a‐b = *Z*: −0.401, *p*: 0.698 a–c = *Z*: −1.07, *p*: 0.283			

a. Perception of the problem: pretest	19.40	5.61	20.00	19.89	4.84	20.00	*U*: 590.5 *p*: 0.793
b. Perception of the problem: posttest	23.63	2.20	25.00	17.09	7.14	20.00	*U*: 298.0 *p*: 0.000
c. Perception of the problem: follow‐up	22.29	4.03	25.00	19.69	4.24	20.00	*U*: 378.5 *p*: 0.004
Statistical analysis/intragroup comparison	a‐b = *Z*: −4.15, *p*: 0.000 a–c = *Z*: −2.36, *p*: 0.018			a‐b = *Z*: −1.81, *p*: 0.069 a–c = *Z*: −0.088, *p*: 0.930			

a. Knowledge of the causes of climate change: pretest	35.00	10.95	36.00	38.40	6.39	39.00	*U*: 531.0 *p*: 0.331
b. Knowledge of the causes of climate change: posttest	42.11	4.19	45.00	33.80	8.53	36.00	*U*: 285.0 *p*: 0.000
c. Knowledge of the causes of climate change: follow‐up	40.60	6.44	45.00	37.51	6.22	37.00	*U*: 400.0 *p*: 0.011
Statistical analysis/intragroup comparison	a‐b = *Z*: −3.72, *p*: 0.000 a–c = *Z*: −2.73, *p*: 0.006			a‐b = *Z*: −2.49, *p*: 0.013 a–c = *Z*: −0.505, *p*: 0.613			

a. Concern about climate change: pretest	44.83	13.22	50.00	48.31	8.37	53.00	*U*: 544.5 *p*: 0.418
b. Concern about climate change: posttest	52.17	4.74	55.00	38.34	15.29	44.00	*U*: 276.5 *p*: 0.000
c. Concern about climate change: follow‐up	49.94	7.48	53.00	48.26	7.19	50.00	*U*: 530.5 *p*: 0.327
Statistical analysis/intragroup comparison	a‐b = *Z*: −3.03, *p*: 0.006 a–c = *Z*: −1.92, *p*: 0.054			a‐b = *Z*: −3.17, *p*: 0.001 a–c = *Z*: −0.206, *p*: 0.837			

a. Behaviors and expectations regarding policies: pretest	72.94	21.41	80.00	79.66	10.44	83.00	*U*: 560.5 *p*: 0.538
b. Behaviors and expectations regarding policies: posttest	84.74	7.64	90.00	76.31	9.29	73.00	*U*: 363.0 *p*: 0.002
c. Behaviors and expectations regarding policies: follow‐up	82.14	11.37	87.00	78.14	12.67	82.00	*U*: 508.5 *p*: 0.217
Statistical analysis/intragroup comparison	a‐b = *Z*: −2.92, *p*: 0.003 a–c = *Z*: −2.10, *p*: 0.035			a‐b = *Z*: −1.56, *p*: 0.118 a–c = *Z*: −0.248, *p*: 0.804			

*Note: M*, mean (arithmetic mean); *U*, Mann–Whitney *U* test statistic.

Abbreviations: CCAS, Climate Change Awareness Scale; Med, median; Sd, standard deviation; SDim, subdimension.

In the follow‐up assessment, the intervention group showed significantly higher scores than the control group in CCAS total score and in the subscales of Perception of the Problem and Knowledge of the Causes of Climate Change (Table 3). No statistically significant differences were found between the groups in the follow‐up mean scores for the subscales Awareness of Climate Change, Concern About Climate Change, and Behaviors and Expectations Regarding Policies (Table 3). Within the intervention group, no significant difference was observed between the pretest and follow‐up scores on the Concern About Climate Change subscale (Table 3).

In the control group, significant differences were observed between pretest and posttest scores for the total CCAS and the subscales Knowledge of the Causes of Climate Change and Concern About Climate Change, with higher scores at the pretest (Table 3). However, no statistically significant differences were found between pretest and follow‐up scores for the total CCAS or any of its subscales (Table 3).

A statistically significant difference was found among the pretest, posttest, and follow‐up mean scores of CCAS and its subscales in the intervention group (*χ* ^2^ = 20.16, *p* < 0.001). According to these results, the pretest scores for CCAS total and subscales were significantly lower than the posttest scores (*p* < 0.05). Furthermore, the posttest scores for CCAS total and the Awareness of Climate Change subscale were significantly higher than the follow‐up scores (*p* < 0.05) (Table 3). No statistically significant differences were found between the posttest and follow‐up scores of the perception of the problem, knowledge of the causes of climate change, concern about climate change, and behaviors and expectations regarding policies subscales in the intervention group (Table 4).

**TABLE 4:** Comparison of pretest, posttest, and follow‐up mean scores between the intervention and control groups.

Scales and subdimensions	Intervention group (*n*: 35)				Control group (*n*: 35)			
	Mean	SD	Med.	S.M.	Mean	SD	Med.	S.M.
a. Total CCAS: pretest	203.40	53.95	220.00	1.44	220.43	29.15	236.00	2.03
b. Total CCAS: posttest	243.06	20.87	250.00	2.49	200.89	43.60	208.00	1.80
c. Total CCAS: follow‐up	231.91	32.84	242.00	2.07	219.43	32.71	231.00	2.17
Statistical analysis/intragroup comparison	*χ* ^2^: 20.16, SD: 2, *p*: 0.000 a ⟶ b = *Z*: −3.89, *p*: 0.000 a ⟶ c = *Z*: −2.94, *p*: 0.003 b ⟶ c = *Z*: −2.05, *p*: 0.040 a < b‐a < c‐b > c				*χ* ^2^: 2.60, SD: 2, *p*: 0.272			

a. Awareness of climate change: pretest	31.23	7.73	32.00	1.31	34.17	5.60	34.00	2.00
b. Awareness of climate change: posttest	40.40	4.49	42.00	2.60	35.34	6.46	36.00	1.90
c. Awareness of climate change: follow‐up	36.94	5.94	37.00	2.09	35.83	6.21	35.00	2.10
Statistical analysis/intragroup comparison	*χ* ^2^: 31.09, SD: 2, *p*: 0.000 a ⟶ b = *Z*: −4.64, *p*: 0.000 a ⟶ c = *Z*: −3.53, *p*: 0.000 b ⟶ c = *Z*: −2.75, *p*: 0.006 a < b‐a < c‐b > c				*χ* ^2^: 0.737, SD: 2, *p*: 0.692			

a. Perception of the problem – pretest	19.40	5.61	20.00	1.51	19.89	4.84	20.00	2.21
b. Perception of the problem – posttest	23.63	2.20	25.00	2.41	17.09	7.14	20.00	1.77
c. Perception of the problem – follow‐up	22.29	4.03	25.00	2.07	19.69	4.24	20.00	2.01
Statistical analysis/intragroup comparison	*χ* ^2^: 18.21, SD: 2, *p*: 0.000 a ⟶ b = *Z*: −4.15, *p*: 0.000 a ⟶ c = *Z*: −2.36, *p*: 0.018 b ⟶ c = *Z*: −1.60, *p*: 0.108 a < b‐a < c				*χ* ^2^: 3.82, SD: 2, *p*: 0.148			

a. Knowledge of the causes of climate change: pretest	35.00	10.95	36.00	1.51	38.40	6.39	39.00	2.10
b. Knowledge of the causes of climate change: posttest	42.11	4.19	45.00	2.37	33.80	8.53	36.00	1.81
c. Knowledge of the causes of climate change: follow‐up	40.60	6.44	45.00	2.11	37.51	6.22	37.00	2.09
Statistical analysis/intragroup comparison	*χ* ^2^: 18.23, SD: 2, *p*: 0.000 a ⟶ b = *Z*: −3.72, *p*: 0.000 a ⟶ c = *Z*: −2.73, *p*: 0.006 b ⟶ c = *Z*: −1.16, *p*: 0.244 a < b‐a < c				*χ* ^2^: 2.00, SD: 2, *p*: 0.368			

a. Concern about climate change: pretest	44.83	13.22	50.00	1.60	48.31	8.37	53.00	2.14
b. Concern about climate change: posttest	52.17	4.74	55.00	2.43	38.34	15.29	44.00	1.73
c. Concern about climate change: follow‐up	49.94	7.48	53.00	1.97	48.26	7.19	50.00	2.13
Statistical analysis/intragroup comparison	*χ* ^2^: 14.42, SD: 2, *p*: 0.001 a ⟶ b = *Z*: −3.03, *p*: 0.006 a ⟶ c = *Z*: −1.92, *p*: 0.054 b ⟶ c = *Z*: −1.84, *p*: 0.065 a < b‐a < c				*χ* ^2^: 4.40, SD: 2, *p*: 0.110			

a. Behaviors and expectations regarding policies: pretest	72.94	21.41	80.00	1.67	79.66	10.44	83.00	2.06
b. Behaviors and expectations regarding policies: posttest	84.74	7.64	90.00	2.31	76.31	9.29	73.00	1.79
c. Behaviors and expectations regarding policies: follow‐up	82.14	11.37	87.00	2.01	78.14	12.67	82.00	2.16
Statistical analysis/intragroup comparison	*χ* ^2^: 8.52, SD: 2, *p*: 0.014 a ⟶ b = *Z*: −2.92, *p*: 0.003 a ⟶ c = *Z*: −2.10, *p*: 0.035 b ⟶ c = *Z*: −1.45, *p*: 0.147 a < b‐a < c				*χ* ^2^: 2.78, SD: 2, *p*: 0.2.49			

*Note:*​ Bonferroni correction applied in post hoc comparisons for within‐group repeated measurements. *χ* ^2^: Friedman test. The nonparametric equivalent of one‐way repeated‐measures ANOVA; *Z*: Wilcoxon signed‐rank test used for pairwise comparisons. M: mean.

Abbreviations: CCAS, Climate Change Awareness Scale; Med, median; SD, standard deviation; S.M., standard error of the mean.

In the control group, no statistically significant within‐group changes were observed across time points (pretest, posttest, and follow‐up) (Table 4). As shown in Table 3, some small differences were observed in between‐group comparisons, which reflect only the median differences between independent groups.

## 4. Discussion

This study aimed to evaluate the effectiveness of a nurse‐led climate change awareness training program for nurse managers. Data were collected at three time points: pretest, posttest, and 1‐month follow‐up. Before the intervention, participants’ self‐reported knowledge level about climate change—measured on a 0–10 scale—was found to be 4.42 ± 2.66, indicating a relatively low level of knowledge. This finding highlights the need for educational interventions designed to enhance nurses’ understanding and awareness of climate change.

The results demonstrated statistically significant differences among the pretest, posttest, and follow‐up scores for CCAS and its subscales in the intervention group, with posttest scores significantly higher than pretest scores. Furthermore, when compared with the control group, nurse managers in the intervention group showed significant improvements in total and all subscale scores awareness of climate change, perception of the problem, knowledge of causes, concern about climate change, and behaviors and expectations regarding policies at the posttest assessment. These results demonstrate that targeted climate change training conducted by nurses increases nurse managers’ awareness of health risks associated with climate change. This offers important implications for nurse managers to shape their leadership and managerial roles in light of these risks. These findings are consistent with previous studies conducted in various populations, including nurses working in rural areas and nursing and midwifery students. The literature supports that educational interventions are effective in increasing awareness, attitudes, and knowledge related to climate change [13, 14, 17, 24].

In this study, the posttest and follow‐up mean scores for CCAS total and the subscales Perception of the Problem and Knowledge of the Causes of Climate Change were significantly higher in the intervention group compared with the control group. This finding indicates that the educational intervention had a positive effect on nurses’ awareness and knowledge regarding climate change. However, no significant differences were observed between the groups in the follow‐up measurements for the subscales Awareness of Climate Change, Concern About Climate Change, and Behaviors and Expectations Regarding Policies. This suggests that longer‐term or reinforcement‐based educational interventions may be required to sustain improvements in these dimensions.

The literature highlights that attitudes toward environmental sustainability are influenced not only by knowledge level but also by motivation, concern, and behavioral tendencies [16]. Indeed, a large‐scale study conducted in Germany with 2066 participants found that as environmental and climate‐specific knowledge increased, anxiety about climate change decreased [25]. This finding suggests that knowledge‐based education alone may not always lead to emotional or behavioral change; therefore, educational programs should be designed to promote not only cognitive gains but also emotional awareness and behavioral engagement.

In our study, although the intervention group’s CCAS posttest scores slightly declined at the follow‐up assessment, they remained higher than the pretest levels. This finding suggests that the educational intervention produced a strong short‐term effect, but its impact diminished partially over time. The literature similarly emphasizes that the long‐term retention of educational effects can be sustained through regular reinforcement and institutional support [26, 27]. Therefore, climate change awareness training should not be a one‐time intervention but rather an integral, ongoing component of in‐service education programs.

The absence of significant changes in the control group over time suggests that the improvements in the intervention group were associated with the educational program. This finding supports the notion that knowledge about climate change does not increase spontaneously but requires structured and targeted training efforts. Leffers and Butterfield [7] reported that although nurses recognize climate change as a health priority, many lack sufficient knowledge on the subject. Consequently, the literature consistently highlights the need to systematically integrate environmental and climate‐related content into nursing curricula to prepare nurses to respond effectively to environmental and climate‐based health threats [28, 29].

The findings of this study show that the training program was associated with positive changes in nurse managers’ overall awareness of climate change and also had positive effects on their perceptions of the issue, their knowledge of the causes of climate change, their levels of concern, their behaviors, and their expectations regarding policies. These findings are particularly important because they suggest that nurses perceive climate change not only as an environmental problem but increasingly as a public health problem. Similarly, a recent study conducted among nurses working at a university hospital examined levels of climate change awareness and associated factors. The results showed that most nurses recognized climate change as a global problem and reported having some knowledge on the subject. However, many nurses expressed uncertainty about their leadership role in educating the public on climate‐related issues and emphasized the need for further education and institutional support in this area [30].

### 4.1. Implications for Nursing Policy and/or Health Policy

The findings of this study demonstrate that awareness training on the relationship between climate change and health is effective for nurse managers, and even short‐term, structured training interventions can yield significant gains. These results suggest that nursing education and in‐service training policies should be systematically strengthened with climate change and health‐focused content. Nurse managers play a key role in translating institutional policies into daily clinical practice, and increasing their awareness of climate‐related health risks can directly impact decision‐making, resource allocation, and staff training. Integrating climate and health‐focused training into routine in‐service training programs can enable nurse leaders to promote sustainable practices, increase preparedness for climate‐related health threats, and foster a culture of environmental responsibility within healthcare institutions. At the institutional level, increasing the knowledge and awareness of nurse managers can support the adoption of climate‐friendly policies, including sustainable resource use, emergency preparedness, and risk mitigation strategies. At the national level, incorporating climate change and health issues into undergraduate and graduate nursing curricula can contribute to the early development of nurses’ competencies in this area. Furthermore, recognizing climate‐related health impacts as priority areas in health policy and supporting nurse leaders in active leadership roles can contribute to the development of long‐term and sustainable health systems.

## 5. Limitations

A limitation of this study is that randomization was performed at the hospital level rather than at the individual participant level, which may limit control over unmeasured institutional differences. First, the relatively short follow‐up period limits the ability to assess the long‐term sustainability of the intervention’s effects. Second, data were collected through self‐reported instruments, which may have introduced response or social desirability bias. Third, the study sample consisted of nurse managers from two public hospitals in a single metropolitan area, which may limit the generalizability of the results to other contexts. Additionally, although the achieved sample size provided adequate power to detect medium‐to‐large effects, smaller effects may not have been captured due to limited statistical power. Finally, complete blinding was not feasible because of the nature of the educational intervention.

## Funding

No funding was received for this manuscript.

## Conflicts of Interest

The authors declare no conflicts of interest.

## Data Availability

The data that support the findings of this study are not publicly available due to ethical and privacy restrictions related to participant confidentiality. However, data are available from the corresponding author upon reasonable request, subject to approval by the relevant ethics committee.
